# Seasonal Variations in Soil Microbiota Profile of Termite (*Syntermes wheeleri*) Mounds in the Brazilian Tropical Savanna

**DOI:** 10.3390/microorganisms8101482

**Published:** 2020-09-27

**Authors:** Helena Ipe Pinheiro Guimaraes, Renata Henrique Santana, Rafaella Silveira, Otavio Henrique Bezerra Pinto, Betania Ferraz Quirino, Cristine Chaves Barreto, Mercedes Maria da Cunha Bustamante, Ricardo Henrique Krüger

**Affiliations:** 1 Cellular Biology Department, University of Brasilia, Campus Universitário Darcy Ribeiro, Brasília D.F. 70910-900, Brazil; helenaipeg@gmail.com (H.I.P.G.); otaviohenriquebp9@gmail.com (O.H.B.P.); 2 Brazilian Federal Institute, Campus Planaltina, Brasília D.F. 70910-900, Brazil; renata.henrique@ifb.edu.br; 3 Embrapa-Agroenergy, Parque Estação Biológica (PqEB), Genetics and Biotechnoloy Laboratory, PqEB s/nº, Brasília D.F. 70770-901, Brazil; rafaella_silveira@hotmail.com (R.S.); betania.quirino@embrapa.br (B.F.Q.); 4 Genomic Sciences and Biotechnology Graduate Program, Universidade Católica de Brasília, Brasilia D.F. 70790-160, Brazil; cristineb@ucb.br; 5 Department of Ecology, Biological Sciences Institute, University of Brasilia, Brasilia D.F. 70910-900, Brazil; mercedes@unb.br

**Keywords:** termite, mound, soil, Cerrado, soil microbiology, environmental microbiology

## Abstract

Eusocial animals, such as the termites, often build a nest-like structure called a mound that provides shelter with stable internal conditions and protection against predators. Termites are important components of the Brazilian Cerrado biota. This study aimed to investigate the bacterial community composition and diversity of the *Syntermes wheeleri* termite-mound soil using culture-independent approaches. We considered the vertical profile by comparing two different mound depths (mound surface and 60 cm) and seasonality with samplings during the rainy and dry seasons. We compared the mound soil microbiota to the adjacent soil without the influence of the mound to test the hypothesis that the Cerrado soil bacterial community was more diverse and more susceptible to seasonality than the mound soil microbiota. The results support the hypothesis that the Cerrado soil bacterial community is more diverse than the mound soil and also has a higher variability among seasons. The number of observed OTUs (Operational Taxonomic Units) was used to express bacterial richness, and it indicates that soil moisture has an effect on the community distribution and richness of the Cerrado samples in comparison to mound samples, which remain stable across seasons. This could be a consequence of the protective role of the mound for the termite colony. The overall community taxonomic profile was similar between soil samples, especially when compared to the taxonomic composition of the *Syntermes wheeleri* termite’s gut, which might be explained by the different characteristics and functionality between the soil and the gut microbial community.

## 1. Introduction

Cerrado is the richest tropical savanna in the world [1]. It represents 24% of the Brazilian territory and it is the second largest biome after the Amazon rainforest [2,3]. The Cerrado soils are predominantly Oxisols characterized by their acidity, red color, low nutrient concentrations, and high clay content and aluminum concentration [3,4,5]. This biome is characterized by dry winters and rainy summers, with markedly seasonal rainfall having a great impact on the soil microbiology function [6,7].

Termite colonies are distributed in the Cerrado according to its niche preferences, such as wood and organic matter abundance [8]. Termite nests have complex structures, and may include subterranean galleries [9]. Termite mound walls are different from the subterranean nest, and provide stability to the community during the rainy season [10]. The presence of the termites in the Cerrado soil is associated with higher pH values, Calcium (Ca), Phosphorus (P), and Potassium (K) concentrations and lower Aluminum (Al) concentrations [11].

Soil physical and chemical parameters are significant determinants of the microbial community profile [12]. The portion of the ecosystem under the influence of the termite colony is called the termitosphere, and it has unique characteristics such as different soil texture and organic matter content when compared to the adjacent soil [13]. Some termite species are able to use the thermal proprieties of the mound to buffer temperature variations in hot and dry areas [14]. Microbial activity is more dependent on the vegetation and seasonality at soil surface (0–5 cm), while with increased profile depth, the microbial community responds more to soil characteristics [6]. The soil microbiota is responsible for 80% of the total processes that occur in the soil, such as carbon and nitrogen cycles [15,16].

Termites are eusocial animals, meaning they have a strict work distribution system according to castes [17]. In fact, the entire colony can be considered one superorganism divided according to its functions: reproduction (alates, kings, and queens), feeding and structure (workers), defense (soldiers), and protection and homeostasis (the mound) [17]. Workers are responsible for constructing the mound and foraging for food [18]. The use of soil for mound construction and consumption was the most important evolutionary event for the termites, and overlapped with the loss of protists which were substituted by bacterial symbionts in the gut of Termitidae termites [18].

The Termitidae family is part of the higher termite clade. It is the most prominent termite family in the tropical region, comprising most of the endemic species in the Cerrado [19]. The *Syntermes* genus includes termites from the subfamily Nasutermitinae, family Termitidae, which are widely distributed in the tropical savannas and are characterized by large size, subterranean mounds, and litter-feeding habits [8]. The absence of flagellates in the higher termite’s gut corresponds to the compartmentalization of the hindgut, which improves fermentation processes in this area [17]. Previous studies showed that the microbiota of the intestinal region and mound soil of the *Syntermes* termites are very different, indicating a higher prevalence of Actinobacteria and Proteobacteria in the mound. In contrast, Firmicutes and Spirochaetes are prevalent in the gut [18,20].

The main objective of this work was to characterize the soil’s bacterial community associated with mounds of the *Syntermes wheeleri* termite and compare it with the Cerrado soil microbiota. We collected the mound soil at two different depths (0–10 cm and 50–60 cm) and in different seasons (dry and rainy). We tested the following hypothesis: (i) the mound soil has lower bacterial diversity than the Cerrado soil, and (ii) seasonality does not affect bacterial richness of the mound soil.

## 2. Materials and Methods

### 2.1. Study Site and Sample Collection

**Figure 1:**
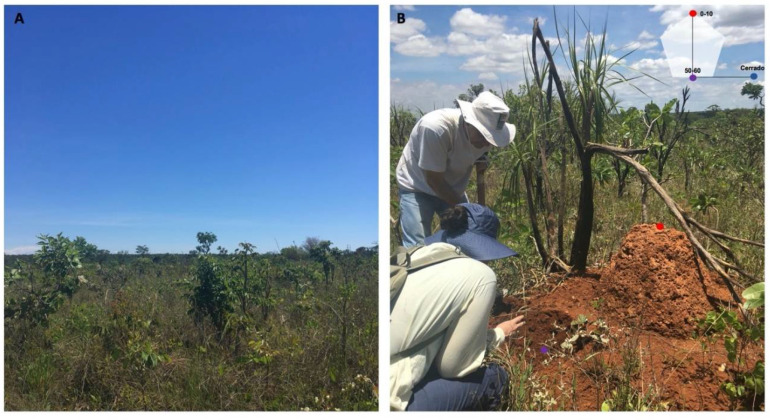
Site overview and sample collection. (**A**) *Campo sujo* in the IBGE (Instituto Brasileiro de Geografia e Estatística) Ecological Reserve during the dry season, and (**B**) *Syntermes wheeleri* termite mound in the *campo sujo* during the dry season. The red circle indicates the collection of the 0–10 cm mound surface soil samples, the purple circle indicates the collection of the 50–60 cm mound soil samples, and the blue circle indicates the collection of the control samples, or Cerrado samples, also at a depth of 50–60 cm within the soil.

**Figure 2:**
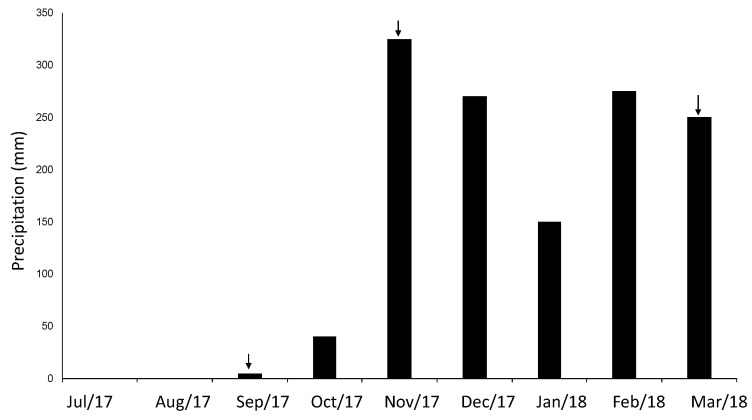
Monthly precipitation data from the Brazilian National Meteorology Institute from July 2017 to March 2018 in Brasilia. Arrows indicate the months in which soil was collected: the dry season in September 2017, transition in November 2017, and late rainy season in March 2018.

We conducted the study in a protected Cerrado area managed by the Brazilian Institute of Geography and Statistics (RECOR Ecological Reserve), Brasilia, Brazil (15°56′54.6″ S; 47°52′11.7″ W). The control soil and the mound soil were collected in a *campo sujo*, a Cerrado vegetation type characterized by the predominance of the herbaceous layer with scattered shrubs and trees (Figure 1A). The *Syntermes wheeleri* termite species was identified by its soldier caste, and mound soil was collected at two different depths: at the mound surface (0–10 cm) and at a depth interval of 50–60 cm (mound soil) (Figure 1B). Control soil was collected at a distance of 100 m from the mound at the depth interval of 50–60 cm. Samples were collected in triplicates at different periods to evaluate seasonal effects: late dry season (September 2017), late rainy season (March 2018), and early rainy season (transition) (November 2017) (Figure 2). Soil was sieved using a 2 mm mesh in situ and stored in plastic bags on ice. After arrival at the laboratory, it was stored at −80 °C until DNA extraction.

### 2.2. Physico-Chemical Soil Parameters

Soil pH was measured after shaking it with distilled water using a HI 2221 Calibration Check pH/ORP calibrated potentiometer (HANNA instruments, Rhode Island, USA). Nitrate and ammonium measurements were performed using a Shimadzu UV-1203 spectrometer (Shimadzu, Kyoto, Japan) at wavelengths of 218, 228, 254, and 280 nm for the nitrate, and with Nessler reagent for ammonium [21]. Soil gravimetric water content was determined after drying it for 72 h at 105 °C [21]. Available P and K were extracted with Mehlich 1, while Ca, Mg (Magnesium), and Al were extracted with KCl 1M. Organic matter (OM) was determined by the Walkley-Black method. These analyses were performed following the EMBRAPA (Brazilian Agricultural Research Corporation) protocols [22].

### 2.3. Soil DNA Extraction and 16S rRNA Gene Library

Genomic DNA was extracted according to the Griffiths et al. protocol [23] with 5% hexadecyltrimethylammonium bromide (CTAB) and quantified with Qubit BR. DNA quality was verified on 1% agarose gel. To describe the soil bacterial community, 27 DNA samples were sent to Macrogen, South Korea. The company constructed a library of the V3-V4 portion of the16S rRNA gene (Bakt_341F: 5′-CCTACGGGNGGCWGCAG-3′ Bakt_805R: 5′-GACTACHVGGGTATCTAATCC-3′), and sequencing was performed in an Illumina MiSeq platform (paired ended, 300 bp, 100 k reads/sample) and results were delivered in the fastq format. Sequencing quality was analyzed using the FASTQC program (0.11.8) (Babraham Institute, Cambridge, UK) [24].

Bioinformatics and statistical analysis. Raw sequences were previously treated by the bbduk program in the BBTools package for Nextera adaptors and Phix sequences removal (BBMap–Bushnell B.–sourceforge.net/projects/bbmap/). The treated sequences were then imported into the QIIME2 2019.7 program (University of Colorado, USA) [25], and the pipeline was executed for pair-ended sequences with the following steps: primer removal [26], quality filtering [27], dereplication, Operational Taxonomic Unit (OTU) 97% clusterization and chimera removal [28], and taxonomy assignment using the SILVA (2018) database for bacteria [29]. To compare the bacterial community among soils, principal component analysis (PCoA) based on Weighted Unifrac distance was calculated. We performed a rarefaction curve and evaluated microbial richness by observed OTUs number (Operational Taxonomic Unit), Pielou and Shannon indexes, which were obtained using the QIIME2 program. To examine differences between predicted gene abundances from functional inference analysis, an analysis of variance (ANOVA) or the Kruskal-Wallis test were performed with the Prism program (8.0.2) (GraphPad software, California, USA). Normality was tested with Shapiro test. A Permutational Multivariance Analysis of Variance (PERMANOVA) was carried out using Rstudio (1.456–© 2009–2018 RStudio, Inc., Boston, MA, USA) to evaluate the differences between bacterial community in the soil samples. Non-Metric Multidimensional Scaling (NMDS) based on Bray–Curtis dissimilarity and Weighted Unifrac PCoA were obtained in Rstudio with vegan [30] and phyloseq [31] packages.

Additionally, functional inferences based on 16S rRNA gene data was performed with a PICRUST2 [32] in QIIME2. The enzyme-encoding gene abundances with their respective metabolic pathways were assigned by Enzyme Commission numbers (EC numbers) and the Kyoto Encyclopedia of Genes and Genomes (KEGG). Only genes assigned to the Nitrogen metabolism were evaluated. Each EC number was confirmed by the MetaCyc Metabolic Pathway database [33]. The functional prediction output was used as an input file on the Prism software. We performed ANOVA with Tukey’s post hoc test to test the differences in enzyme-encoding gene abundances between soil samples during the dry season. The dry season was particularly observed because we found higher microbial diversity during this period (see Section 3). In addition, the majority of recent studies in Cerrado soils have been shown a higher microbial diversity in the dry season. Before statistical analysis, data were tested for normality by the Shapiro test.

## 3. Results

### 3.1. Comparison of Bacterial Community of Mound and Control Soil in the Dry Season

**Figure 3:**
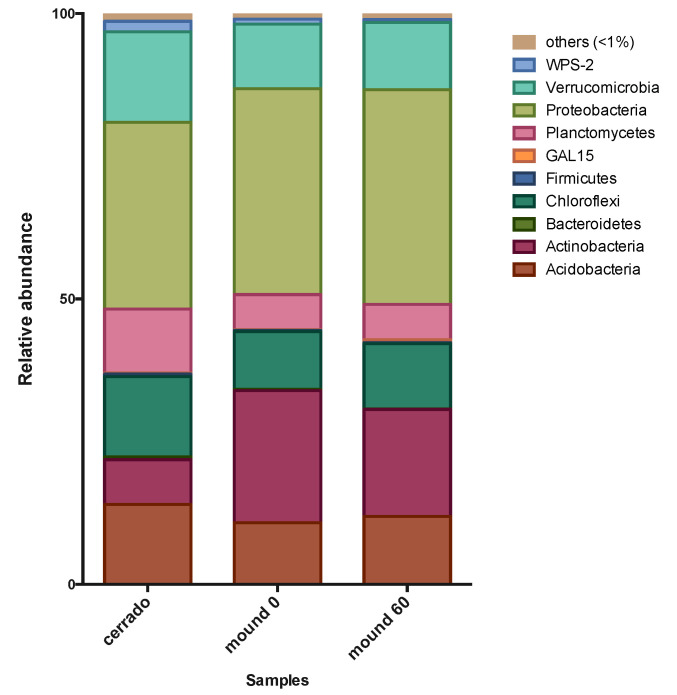
Microbial phyla profile of the dry season for the Cerrado (50–60 cm), mound surface (0–10 cm), and mound soil (50–60 cm) samples in 2017. The graph shows the relative abundance of the most abundant bacterial phyla. Values presented are the mean from the Cerrado, mound surface, and mound soil triplicates in the dry season.

The most abundant phyla, Acidobacteria, Actinobacteria, Chloroflexi, Planctomycetes, Proteobacteria, Verrucomicrobia, and WPS-2, were common in the control soil, mound surface and mound 60 cm soil samples in the dry season. Actinobacteria and Proteobacteria were significantly more abundant in the mound soil samples than in control samples. Rare phyla were defined as those that represent less than 1% of the total bacterial community, comprising 2% of the control soil total community and 1.5% of the mound soil total community (Figure 3).

### 3.2. Seasonality and Chemical Parameters Effect on Bacterial Distribution in Mound and Control Soil Samples

**Figure 4:**
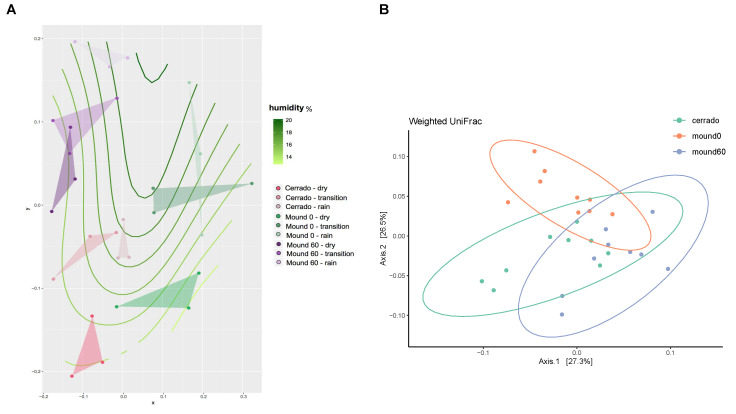
Non-metric dimensional scale analysis from the Cerrado soil samples (50–60 cm), mound surface (0–10 cm), and mound soil (50–60 cm) at the dry, transition, and rainy seasons. (**A**) Non-Metric Multidimensional Scaling (NMDS) of the bacterial community in regard to soil moisture content. Data from the bacterial Operational Taxonomic Unit (OTU) matrix was distributed according to the Bray–Curtis dissimilarity analysis and disposed with its respective soil sample humidity measure. (**B**) The weighted Unifrac matrix analysis displays the sites of the Cerrado soil in green, mound surface in orange, and mound soil in lavender.

**Figure A1:**
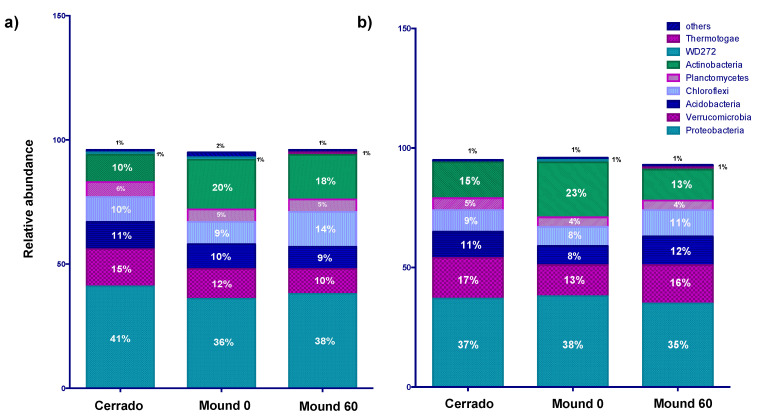
Relative abundance of the bacterial phyla in the rainy season and transition for the Cerrado (60 cm), mound surface (0 cm), and mound soil (60 cm) samples. The samples are shown in the *x* axis and the phyla in the *y* axis for the transition (**a**) and rainy season (**b**). ‘Others’ refer to groups that represent less than 1% of the total bacterial community.

**Figure A2:**
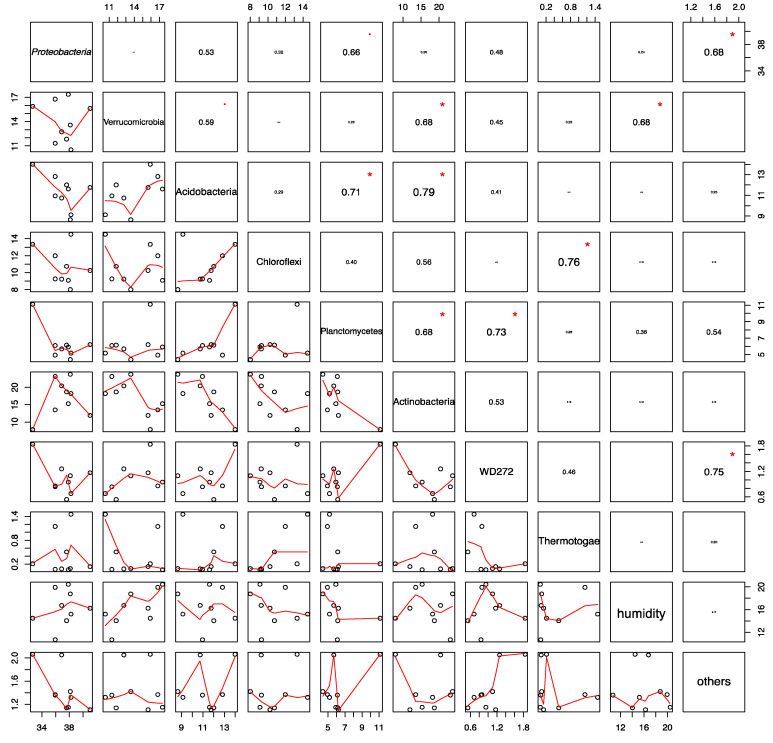
Correlation between the most abundant phyla in the soil and humidity. Linear regression analysis between bacterial phyla and soil moisture content for the Cerrado soil (60 cm depth), mound surface (0 cm), and mound soil (60 cm depth) samples. The numbers shown represent the R value in the linear regression, and the * symbol in red are statistically significant values.

The most abundant phyla among the samples of the late and early rainy season were: Acidobacteria, Actinobacteria, Chloroflexi, Planctomycetes, Proteobacteria, Verrucomicrobia, WD272, and Thermotogae (Figure A1). The season’s influence on the bacterial community was further explored through a Non-Metric Multidimensional Scaling (NMDS) graph, where samples were distributed according to the Bray–Curtis dissimilarity matrix calculated from the OTU (Operational Taxonomic Unit) table for the bacterial community (Figure 4A), although the PERMANOVA analysis of the weighted Unifrac matrix did not significantly differentiate samples in regard to the site of collection (Figure 4B). Verrucomicrobia phylum positively correlates with humidity (Figure A2).

**Figure 5:**
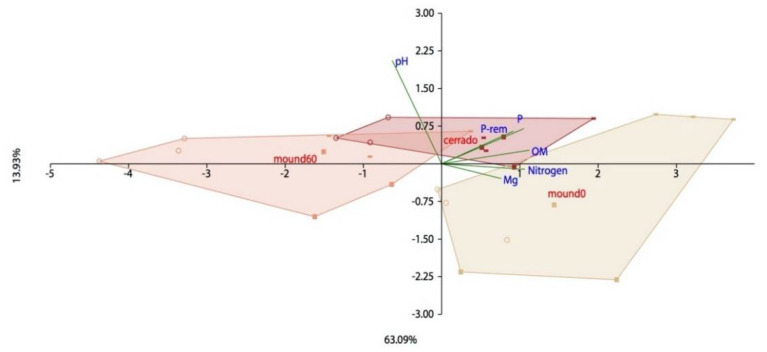
Principal component analysis of the soil chemical parameters for termite mound (mound 0 and mound 60) and Cerrado samples (cerrado) collected in 2017 and 2018. Parameter values are in Table S1. A previous analysis was performed to remove correlated variables, and the remaining parameters are pH, P, P-rem (remaining P), OM (Organic matter), Nitrogen, and Mg. The dry season is represented with a circle, the transition season with a square, and the rainy season with a dash.

Values for N-nitrate, P, moisture, pH, K, Ca, Mg, Al, ammonium, total N, remaining-P, and organic matter are shown in Table S1. Since the seasons did not significantly affect parameter analysis, the PCA aggregates all of the samples for a greater statistical inference, which separates mound soil depths and Cerrado with a 63% of explanation related to soil chemical composition (Figure 5).

### 3.3. Ecological Parameters and Indexes for Soil Samples and Seasons

**Figure 6:**
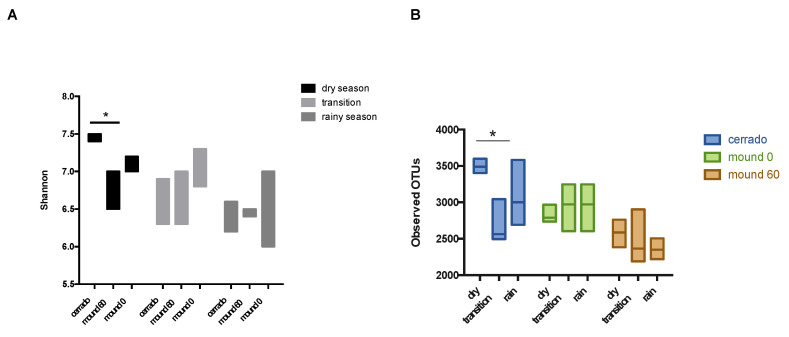
Diversity and richness measures for the microbial community for the Cerrado (50–60 cm), mound surface (0–10 cm), and mound soil (50–60 cm) samples collected in Brasilia, 2017 and 2018. (**A**) Shannon index values for microbial diversity. The dry, transition, and rainy seasons are shown by the grey scale bar. Significant values were considered for *p* value < 0.05 and are indicated with an asterisk ‘*’. (**B**) Analysis of the observed OTU number for each soil sample at the dry, early, and late rainy seasons. Statistically significant values were considered for *p* value < 0.05 and are indicated with an asterisk ‘*’.

**Figure A3:**
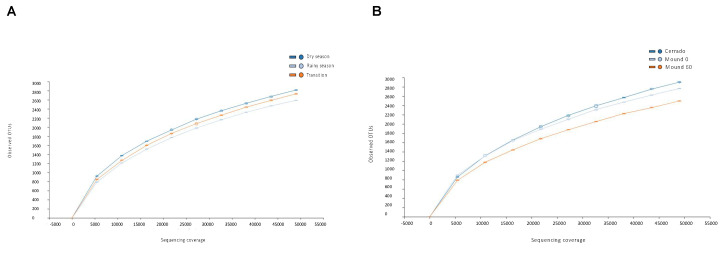
Alpha rarefaction curve of the observed OTUs. (**A**) Cerrado soil bacterial community, collected at different seasons (dry, rainy, and transition) in 2017 and 2018; (**B**) bacterial community in soil samples of the Cerrado (60 cm depth), mound surface (0 cm), and mound soil (60 cm depth) collected in 2017 and 2018.

**Figure A4:**
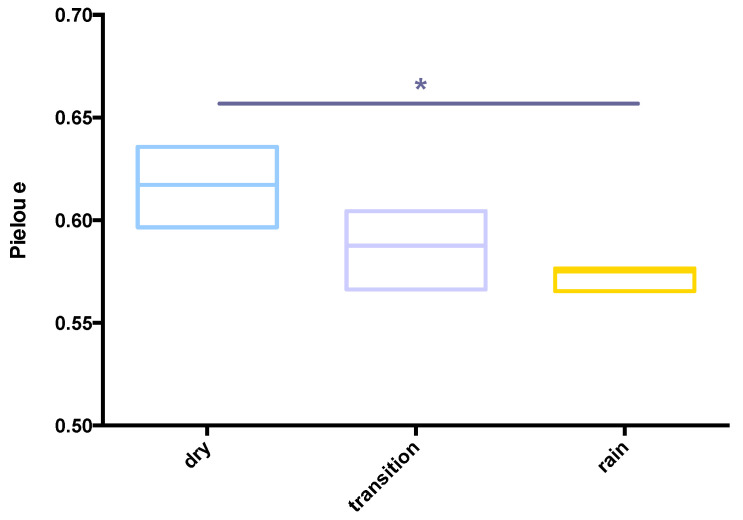
The Pielou number for bacterial equitability in the Cerrado soil (60 cm depth), mound surface (0 cm), and mound soil (60 cm depth) samples collected for the dry, transition, and rainy season in 2017 and 2018. Statistically significant values (*p* < 0.05) are indicated with ‘*’.

The Shannon index indicates that the Cerrado soil samples are more diverse than the mound samples in the dry season (Figure 6A). The OTU (Operational Taxonomic Unit) number for the mound and control samples increases according to the number of sequences in the rarefaction curve. However, it does not reach a plateau, which indicates that the sequencing of samples does not cover the microbiome entirely (Figure A3). The Cerrado soil is the only sample that has a significant difference in the observed OTU number according to seasons, showing a decrease in bacterial richness as the rainy season progresses, while seasonality has no statistical difference for mound surface (0–10 cm) and mound soil (50–60 cm) richness (Figure 6B). The Pielou index, a measure of equitability, did differ between Cerrado and mound soil samples. However, the dry season has a higher Pielou index in comparison to the early and late rainy seasons (Figure A4).

### 3.4. Functional Inference from Taxonomy 

**Figure 7:**
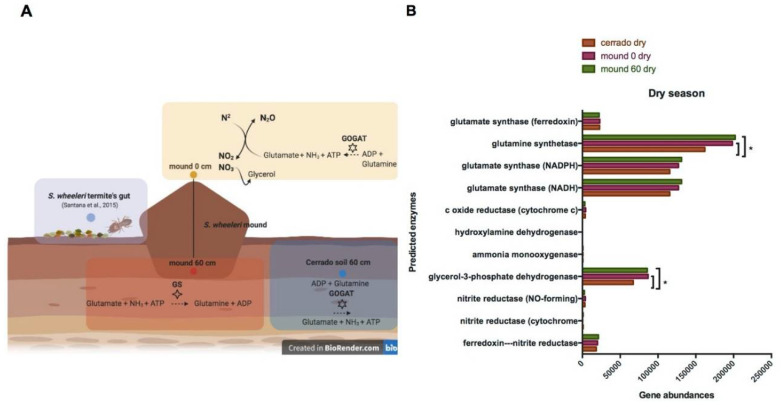
Termite mound soil in the dry season. (**A**) mound samples are shown in orange (0 cm, surface) and red (60 cm depth), and the Cerrado soil samples are in blue in the dry season. The enzymatic reactions that possibly occur in greater abundance at the surface are: nitrogen metabolism—from its fixation until nitrite, nitrate, and glycerol production and the GOGAT pathway. The reverse reaction for glutamine production via GS pathway occurs in greater abundance in the mound 60 cm samples, whereas this pathway is directed to glutamate production in the Cerrado samples via GOGAT. (**B**) Enzyme-encoding genes abundance comparison between soil (50–60 cm) and mound soils (50–60 cm; 0–10 cm) collected in a Cerrado area during the dry season of 2017. The functional prediction based on 16S rRNA gene taxonomic inference in PICRUST2 using the Kyoto Encyclopedia of Genes and Genomes (KEGG) database. Differences in mean proportions (ANOVA, *p* < 0.05) are indicated by asterisks.

**Figure A5:**
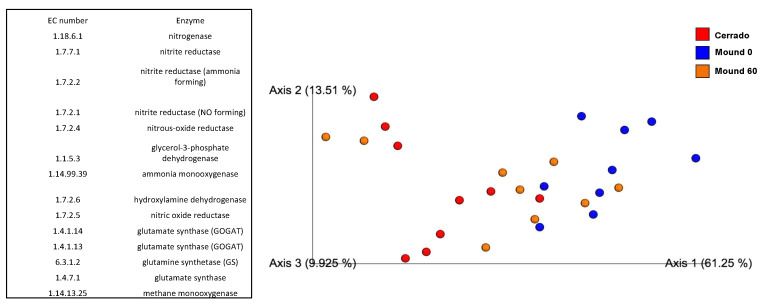
Principal component analysis (PCoA) of the functional inference data from mound soil (60 cm), mound surface (0 cm), and Cerrado soil (60 cm) samples. The principal component coordinates were calculated with the Bray–Curtis dissimilarity matrix and separated the samples in red (Cerrado), blue (mound 0), and orange (mound 60) (Axis–61.25%; Axis 2–13.51%, Axis 3–9.925%). The enzymes shown are common to all samples and participate in the nitrogen cycle according to KEGG database.

Regardless of taxonomical difference, functional prediction analysis allows us to infer the influence of the site or season in microbial processes. Genomic prediction from 16S rRNA gene data was performed for each sample, and the output is a table with all the functional pathways that can be used for a dissimilatory analysis. The final result indicates a possible functional redundancy between samples (Figure A5). Moreover, functional analysis of soil metataxonomic data enables annotation of nitrogen metabolism enzymes (Figure 7A). All 27 samples can potentially fix nitrogen (EC: 1.18.6.1), reduce nitrate to nitrite (EC: 1.7.7.1), perform nitric oxide reduction (EC: 1.7.2.1), produce glycerol precursors (EC: 1.1.5.3), oxidize ammonia to nitrite (EC: 1.14.99.39), and produce glutamine from ammonia and glutamate (EC: 1.4.1.14) (Table S2). The glutamine synthetase (GS, EC: 6.3.1.2) is also present in the samples and possibly more abundant in the mound soil (50–60 cm) during the dry season (Figure 7B).

## 4. Discussion

We compared the bacterial community of termite-mound soil in different seasons to *campo sujo* Cerrado soil, used as a control. The most abundant bacterial phyla found in the soil samples in this study were Proteobacteria, Acidobacteria, Actinobacteria, Verrucomicrobia, Chloroflexi, and Planctomycetes. These phyla are commonly found in soil samples as previously reported [12,16,24,34,35,36]. Most bacterial phyla in soil are rare; nonetheless, this “low abundance” part of the community represents 41% of total 16S rRNA gene sequences and are found in a great variety of soils [35]. Commonly found in the mounds, Actinobacteria have a symbiotic relationship with termites. These bacteria recycle nutrients and produce antimicrobial peptides protecting the nest against foreign pathogens [37,38]. Actinobacteria are also considered to be the second most abundant bacterial phyla in the gut of higher termites and are mostly prevalent on those that feed on humic matter and soil [39]. However, that is not what was observed for the *Syntermes wheeleri* termite gut, which has Firmicutes, Bacteroidetes, and Spirochaetes as the most abundant phyla [20].

Similarly to the termite nest soil, the termite’s gut has been object of study as a distinctive ecological niche for thousands of different bacterial species and phylotypes with a broad set of metabolic activities [40]. These include lignocellulolytic degradation, methanogenesis, CO_2_-reduced acetogenesis, nitrogen fixation, and other activities [40,41]. The termite’s nest taxonomic content is usually significantly different from that of the termite’s gut [18,42]. This could be explained by the different physiological conditions in the termite’s gut (i.e., anoxygenic and alkaline) and the termite’s nest soil (i.e., oxygenic and acidic). Regarding this study specifically, the pH of the *S. wheeleri* termite gut was reported as 9–10 [20] and the Cerrado soil between 5–6. Along with that, structural variations between gut and soil confer different microbial niches [42]. Nonetheless, microbial biomass is inevitably transferred through feces from the termite’s gut to the nest soil, directly impacting the microbial community structure. The work of Minkley and collaborators [43] showed that the bacterial community of the gut of the *Hodotermes mossambicus* termite species is nest-specific, and it is used to discriminate between nestmates and non-nestmates—an ability that is disrupted in antibiotic-treated termites [44].

Proteobacteria is currently the largest bacterial group, and it is also the most diverse. A lot of representatives in this phyla, such as the Rhizobia, are known for their ecological function in the nitrogen cycle, particularly for their ability to interact symbiotically with the plant root system and fixate nitrogen [45]. Ammonia is the primary product of the nitrogen fixation reaction via symbiosis [46] and also a product from glutamate and glutamine reduction via glutamine synthetase and glutamate synthase pathways [47]. Glutamine synthetase is responsible for glutamine production, which is associated to bacterial growth [48,49]. The glutamine synthetase enzyme appears to be more abundant in the mound surface (0–10 cm) soils and mound soil (50–60 cm) than the control soil (50–60 cm) during the dry season. In contrast, the glutamate synthase enzyme, present in all samples in the dry season, is responsible for executing the reverse pathway for production of glutamate [50], an amino acid associated with stress responses by bacteria in acidic environments [51].

Chemical processes and microbial activity are connected in the soil environment [52]. Simultaneous analysis of the bacterial community and soil parameters demonstrated that pH is the most significant factor for microbial composition, diversity, and biomass [53]. Soil is usually more acidic on the surface than deeper within [54], and moisture can alter the chemical composition of Cerrado soil [55]. Silva and collaborators also showed that Verrucomicrobia was the phyla most associated to the rainy season in Cerrado, corroborating results in this work. The dry season has the greater equitability (Pielou index) and bacterial richness (OTU number) in comparison to the other seasons. This phenomenon might be related to the fact that the environment is hostile during the dry season, and no specific group is favored. The more homogenous the community, the more resilient it is to external changes [56]. However, low richness communities can be as productive as high richness communities since the species composition and metabolic activity are important variables to be considered [13,57].

Sequencing techniques allow us to identify bacterial species, revealing the microbiota composition and its relationship with abiotic elements without the need to cultivate microorganisms [12]. Functional inferences based on taxonomy have been able to reconstitute the main functional aspects of a community [58,59,60,61], despite some limitations [32]. As the scientific community produces more information regarding 16S rRNA gene data and metagenomic sequences, a pattern of two gene cores emerged: a large set of non-essential genes or pseudogenes, as well as a set of specific genes selected by the environment [62]. Metagenomic analysis of mound soil demonstrated that it was functionally similar to that of the adjacent soil, with the exception of virulence, pathogenicity, and defense sequences, which are found in the mound and are responsible for nest protection [38]. The abundance of defense genes is often associated to the extensive reservoir of Actinobacteria found in these mounds [37].

## 5. Conclusions

Termites are known as ecosystem engineers due to their ability to modulate the environment through its activities. In this work, the most relatively abundant bacterial phyla are common to soil samples of both Cerrado and mound soils, and they are: Acidobacteria, Actinobacteria, Chloroflexi, Planctomycetes, Proteobacteria, Verrucomicrobia, and WPS-2, which differ from the abundant phyla present in the *Syntermes wheeleri* gut. Interactions between these microbial groups and abiotic elements of the ecosystem have not been studied yet. Diversity and functional redundancy are important aspects of a community that enable the coexistence of different groups that can perform the same process, thus contributing to the ecosystem’s stability. The principal component analysis shows three different soil samples: the Cerrado (50–60 cm), the mound surface (0–10 cm), and the mound soil (50–60 cm). Cerrado soil is considered to be the most diverse among all samples analyzed, according to the Shannon index, confirming the first hypothesis: “The mound-associated soil has a smaller bacterial diversity than the surrounding Cerrado soil”.

Seasons are a relevant variable in the Cerrado biome, having great influence on the soil microbiome. Moisture interferes in the bacterial community structure and distribution in Cerrado and mound soil samples. This can be observed by the number of Operational Taxonomic Units (OTUs), which is significantly reduced as the seasons progress for the Cerrado samples (50–60 cm), implying that the bacterial richness in this soil is more susceptible to seasonal changes than mound soil samples, which corroborates the second hypothesis: “seasonality does not affect the mound-associated soil bacterial richness”. The results presented in this work relate microbial ecology elements of the termite mound soil to abiotic elements present in the Brazilian Cerrado biome. Future studies should address the dynamics of termite activity and soil microbiota.

## Supplementary Materials

The following items are available online at https://www.mdpi.com/2076-2607/8/10/1482/s1, Table S1: Chemical parameters of the soil from the Cerrado (50–60 cm), mound surface (0–10 cm) and mound (50–60 cm depth) samples in the dry, transition and rainy seasons; Table S2: Predicted enzymes of the Nitrogen cycle from the Cerrado (60 cm), mound surface (0 cm) and mound (60 cm) soil samples.
